# Using Matrix-Product States for Open Quantum Many-Body Systems: Efficient Algorithms for Markovian and Non-Markovian Time-Evolution

**DOI:** 10.3390/e22090984

**Published:** 2020-09-04

**Authors:** Regina Finsterhölzl, Manuel Katzer, Andreas Knorr, Alexander Carmele

**Affiliations:** Institut für Theoretische Physik, Nichtlineare Optik und Quantenelektronik, Hardenbergstraße 36, 10623 Berlin, Germany; manuel.katzer@physik.tu-berlin.de (M.K.); andreas.knorr@tu-berlin.de (A.K.); alexander.carmele.1@tu-berlin.de (A.C.)

**Keywords:** quantum spin chains, matrix-product states, open quantum systems, many-body systems, numerical methods, quantum stochastic Schrödinger equation, feedback control

## Abstract

This paper presents an efficient algorithm for the time evolution of open quantum many-body systems using matrix-product states (MPS) proposing a convenient structure of the MPS-architecture, which exploits the initial state of system and reservoir. By doing so, numerically expensive re-ordering protocols are circumvented. It is applicable to systems with a Markovian type of interaction, where only the present state of the reservoir needs to be taken into account. Its adaption to a non-Markovian type of interaction between the many-body system and the reservoir is demonstrated, where the information backflow from the reservoir needs to be included in the computation. Also, the derivation of the basis in the quantum stochastic Schrödinger picture is shown. As a paradigmatic model, the Heisenberg spin chain with nearest-neighbor interaction is used. It is demonstrated that the algorithm allows for the access of large systems sizes. As an example for a non-Markovian type of interaction, the generation of highly unusual steady states in the many-body system with coherent feedback control is demonstrated for a chain length of N=30.

## 1. Introduction

The study of open quantum systems forms one of the main problems of modern physics. Broadly speaking, such a system consists of a microscopic region with quantum coherence which is coupled to an external environment [1,2,3,4], where the interaction leads to decoherence. With the recent development of the ability to control quantum coherence of single particles—which is, for instance, important for the storage and manipulation of quantum information [5]—open quantum systems have received increasing attention in a broad range of fields of physics. Examples include solid state physics, the investigation of trapped atoms [6,7], molecules and ions [8,9], electron transport through quantum dots or other mesoscopic devices [10]; the area of quantum optics, here for example the investigation of photon modes in cavities [11] or impurities coupled to photonic crystals [12]; and photo-synthetic complexes in quantum biology [13]. See [10,14,15,16] for excellent reviews on these topics.

As a special case of open quantum systems, strongly interacting many-body systems also have drawn increasing interest recently, for instance for the purposes of quantum simulation [17]. In particular, quantum spin chains have been the subject of interest due to the rich variety of phenomena arising from the environmental interaction such as phase transitions [18,19,20,21,22,23], quantum transport properties [24,25,26,27,28,29,30,31,32] and entanglement structure [33,34].

In the theory of open quantum systems, one of the methodological tasks is to avoid the integration of the entire system which usually consists of large number of degrees of freedom, thus to trace out the environment and evolve the reduced density matrix of the system $\rho_{s}\left(t\right)$. Here, master equations provide a way to compute this evolution—in particular the Lindblad–Kossakowski form which may be applied for systems where the Markov approximation holds [35]. Other approaches include quantum Langevin equations [36], the input-output formalism using the Heisenberg representation [37] or continuous measurement theory [2].

In case of a quantum many-body system, the reduced Hilbert space grows exponentially with the number of particles involved, often making analytical solutions difficult and numerical approaches very demanding—for instance, solving the full set of differential equations of the reduced density matrix within the master equation framework generally limits the system size to N≈10 where *N* counts the number of qubits of the many-body system.

Another widely used approach is the quantum trajectories method [3,14,38,39]. Instead of propagating the density matrix, it relies on the time evolution of pure states and describes the coupling to the environment with stochastic quantum jumps. Here, system sizes as large as N≈20 qubits are within reach. Clearly, to access larger system sizes, approximative methods are needed which allow for an efficient reduction of the exponentially growing Hilbert space.

In this paper, we present a powerful combination of two tools. We use an alternative approach, the quantum stochastic Schrödinger equation (QSSE) [40,41,42]. Like in the master equation approach, the state vector of the reduced density matrix is evolved in time, while its interaction with the environment is encoded in its dependence on an additional noise with certain statistical properties. We combine this approach with a powerful numerical tensor network method called matrix-product states (MPS), which allows for an efficient reduction of the Hilbert space by truncating the eigenvalues of the decomposed state vector. While this combination has been successfully applied on open two- or few-level systems [43], we present here an efficient application on a many-body system taking into account that initially, the external environment is in the vacuum state and yet uncorrelated with the microscopic region or system. Exploiting on this, we present efficient ways to structure the MPS-architecture both for the case of Markovian as well as for non-Markovian interaction.

To demonstrate the potential of the proposed algorithm, we model the Heisenberg spin-1/2 chain [44] as a paradigmatic many-body system. This model is particularly important as it is analytically solvable [45,46] and forms the backbone to explain experiments in the domain of strongly correlated many-body physics [47,48,49,50,51].

This article is structured as follows. In Section 2, we describe the time evolution with matrix-product states (tMPS) and briefly sketch the broad range of its applications in the Schrödinger as well as in the density matrix picture. In Section 3, we demonstrate the derivation of the numerical basis in the picture of the quantum stochastic Schrödinger equation for systems with Markovian type of interaction and explain the algorithm in detail. Subsequently, we present in Section 4 the application of the algorithm for the more general, yet numerically more complicated case of non-Markovian type of interaction between many-body system and reservoir. Finally, in Section 5, we demonstrate the potential of the algorithm on the paradigmatic example of the Heisenberg spin chain, before we conclude in Section 6.

## 2. Time Evolution with Matrix-Product States

Alongside the above mentioned numerical methods, algorithms based on the description of the wave vector as a matrix-product state (MPS) have firmly established themselves as an important tool for the numerical treatment of quantum physical systems. They were initially used in an analytical context [52,53,54] and their first numerical application took place in the context of the density matrix renormalization group (DMRG) technique for ground state calculations [55,56,57]. In this context, algorithms for the time evolution like the time-dependent density matrix renormalization group (tDRMG), the time-evolving block decimation (TEBD) [58,59,60,61] and the time evolution of the MPS state (tMPS) have been developed. They all perform the same operation, which is the application of a time-evolution operator $U\left(t,t_{0}\right)=\hat{T}\exp\left(-i\int_{t_{0}}^{t}H^{\prime }\left(t^{\prime }\right)dt^{\prime }\right)$ on a state |ψ(t)〉, thus the time evolution is described by the time-dependent Schrödinger equation with

(1) |ψ(t+Δt)〉=U(Δt)|ψ(t)〉.

Here, *H* denotes the Hamilton operator of the system which is chosen to be time-independent here, *i* is the imaginary unit, *ℏ* the reduced Planck constant, and Δt one time step. They only differ in one aspect as both tDRMG and TEBD make use of variational methods while tMPS does not [56], which is why we make use of the latter in this paper.

Central to these algorithms is the expansion of the state vector coefficient into a product of tensors using the singular value decomposition. In this form, the entanglement between the subsystems is accessible in form of the singular values which provides a valid truncation of the Hilbert space by setting those below a certain threshold to zero. Combining this technique with the Suzuki-Trotter decomposition [62,63] enables the approximation of U(t) as sparse matrix exponentials [56,58,64]. Details are given in Section 3 and Section 4. Note however that without a truncation of the Hilbert space, this numerical method is in principle exact—thus, given the numerical resources, it is always possible to find a Δt small enough to describe an exact evolution of the state vector.

Despite the fact that tMPS has been developed for the application on pure states only, it has been successfully applied on incoherent dynamics of mixed states of open many-body systems described in the density matrix picture with the Markovian master equation [65,66,67,68,69,70,71,72,73] and recently even on few-level systems with non-Markovian [74] dynamics. However, the scaling of the Hilbert space with $4^{N}$—instead of $2^{N}$ in Schrödinger space—again imposes a strong disadvantage for larger system sizes *N*. Additionally, the readout of expectation values is limited [67].

For all coherent dynamics with pure states, however, the more efficient Schrödinger picture allows for the full access of expectation values and may be applied on systems with Markovian as well as non-Markovian dynamics, which has already successfully been demonstrated for open few-level systems [43,75,76,77]. For this, the picture of the quantum stochastic Schrödinger equation serves as a numerical basis. However, as we will demonstrate further below, this includes the need to explicitly describe the time-dependent state of the reservoir, and with this, the entanglement between system and reservoir again leads to a very unfortunate scaling with *N*.

In the following, we present an algorithm which allows for an efficient time evolution of Markovian as well as non-Markovian dynamics as it overcomes these difficulties imposed by information preservation and entanglement growth. It thus enables the access to larger systems than with other numerical methods like solving the master equation or quantum jump simulations.

## 3. Modeling Markovian System–Reservoir Interaction

### 3.1. Model

**Figure 1:**
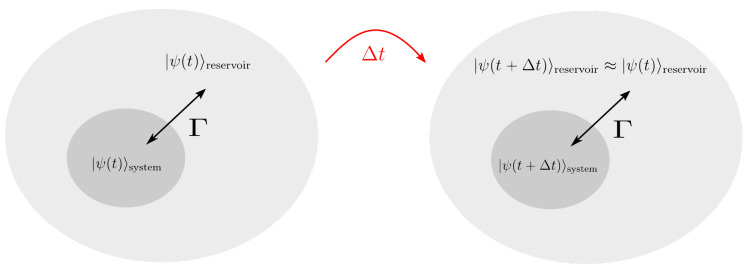
Sketch of an open quantum system with Markovian type of interaction. The total system consists of a microscopic region ${|\psi (t)\rangle }_{\mathrm{sys}}$ which couples to its surrounding environment or reservoir ${|\psi (t)\rangle }_{\mathrm{res}}$ with a coupling strength Γ. During time evolution for one time step Δt, the Markov approximation requires that the reservoir recovers instantly from the interaction and relaxes again into its previous state, thus ${|\psi (t+\Delta t)\rangle }_{\mathrm{res}}={|\psi (t)\rangle }_{\mathrm{res}}$.

As the QSSE serves as numerical basis, cf. Section 3.2, our algorithm is applicable e.g., for modeling the dynamics of systems coupled to a bosonic reservoir consisting of harmonic oscillators, for instance to an electromagnetic field, see Figure 1. Two further assumptions must be made. First, in order apply the rotating frame approximation, the separation of time scales must hold, thus $\omega_{0}\gg \Gamma$. Secondly, to describe the reservoir as a driving field with white noise properties, it must be described in the weak coupling limit [40]. Examples are radiatively damped atoms or a cavity mode coupled to a mode continuum via a partially transmitting mirror.

The total Hamiltonian $H_{\mathrm{tot}}$ consists of the (many-body) system, reservoir and interaction parts. It reads (with ℏ=1):

(2) $$H_{\mathrm{tot}}=H_{\mathrm{sys}}+H_{\mathrm{res}}+H_{\mathrm{int}},$$

with the Hamiltonian of the free evolution of the bosonic mode continuum defined as

(3) $$H_{\mathrm{res}}=\int d\omega \omega b^{†}\left(\omega \right)b\left(\omega \right).$$

Here, $b^{\left(†\right)}\left(\omega \right)$ creates/annihilates a bosonic excitation of energy ω in interaction with the system. They obey the commutation relations:

(4) $$\left[b\left(\omega \right),b{\left(\omega^{\prime }\right)}^{†}\right]=\delta \left(\omega -\omega^{\prime }\right)$$

Please note that for simplicity, only one reservoir is assumed; however, the derivation is easily extended to the generalization of many reservoirs in interaction with a many-body system. The interaction part in the rotating wave approximation describes a linear system-field coupling:

(5) $$H_{\mathrm{int}}=\int d\omega \left(g_{0}b^{†}\left(\omega \right)c^{-}+h.c.\right)$$

with the frequency-independent coupling $g_{0}=\sqrt{\Gamma /2\pi }$. Here, *c* is a system operator whose free evolution we assume to be described by $c\left(t\right)=e^{-i\omega_{0}t}$, governed by the system frequency $\omega_{0}$. For $H_{s}$, no further specifications are being made. Please note that with these assumptions, Equation (2) models the Lindblad master equation of the form (ℏ=1):

(6) $$\begin{array}{cc} \frac{d}{\mathrm{dt}}\rho_{s}\left(t\right)\; & =-i\left[H_{sys},\rho \left(t\right)\right]+\Gamma D\left[\sigma_{N}^{-}\right]\rho \left(t\right) \end{array}$$

with the reduced density matrix $\rho_{s}\left(t\right)$ and the Lindblad superoperator $D\left[J\right]\rho =2J\rho J^{†}-J^{†}J\rho -\rho J^{†}J$.

However, for the proposed algorithm, we remain in the Schrödinger picture. The deterministic evolution is determined by the Schrödinger equation

(7) $$i\frac{d}{\mathrm{dt}}|\psi (t)\rangle =H|\psi (t)\rangle .$$

### 3.2. Quantum Stochastic Schrödinger Equation (QSSE)

To achieve a basis for our numerical systems, we go into the picture of the quantum stochastic Schrödinger equation. Instead of tracing out the reservoir’s degrees of freedom, we remain in the Schrödinger picture and use a time discrete basis which includes the interaction with the reservoir at one time step with a stochastic, time-stroboscopic description [40,43,77].

We demonstrate the derivation of the basis for the case of Markovian system–reservoir interaction. Thus, one (or more) sites of the quantum system are subject to dissipation, i.e., they are coupled to a vacuum reservoir with vacuum input for every time step. For this, first we transform Equation (2) into the rotating frame defined by its freely evolving part using the unitary transformation with

(8) $$H^{\prime }=U_{1}HU_{1}^{†}-iU_{1}\partial_{t}U_{1}^{†}$$

where the unitary operator $U_{1}$ is defined as:

(9) $$U_{1}=\exp\left[it\left(\sum_{i=0}^{N}\omega_{0}c_{i}^{†}c_{i}+\int d\omega \omega b^{†}\left(\omega \right)b\left(\omega \right)\right)\right]$$

This yields the transformed Hamiltonian $H^{\prime }\left(t\right)$:

(10) $$\begin{array}{cc} H^{\prime }\left(t\right)=\; & \int d\omega \left(g_{0}c^{†}b\left(\omega \right)e^{-i(\omega -\omega_{0})t}+h.c.\right) \end{array}$$

We define time-dependent bath operators $b^{\left(†\right)}\left(t\right)$ with

(11) $$b\left(t\right)=\frac{1}{\sqrt{2\pi }}\int_{-\infty }^{\infty }d\omega b\left(\omega \right)e^{-i(\omega -\omega_{0})t}$$

where taking the integral from −∞ to +∞ is called the narrow bandwidth approximation. This means that the commutation relations take on the form of a δ-function:

(12) $$\left[b\left(t\right),b^{†}\left(t^{\prime }\right)\right]=\delta \left(t-t^{\prime }\right),$$

and with this, the $b{\left(t\right)}^{\left(†\right)}$ model white noise. The next step is to introduce time discrete quantum noise operators which include the interaction with the reservoir at one time step with a stochastic, continuous description [43,77]:

(13) $$\begin{array}{c} \Delta B^{\left(†\right)}\left(t_{k}\right)=\int_{t_{k}}^{t_{k+1}}dt^{\prime }b^{\left(†\right)}\left(t^{\prime }\right) \end{array}$$

The following commutation relations hold:

(14) $$\begin{array}{cc} {}[B\left(t_{k}\right), & B^{†}\left(t_{j}\right)]=\int_{t_{k}}^{t_{k+1}}dt\int_{t_{j}}^{t_{j+1}}dt^{\prime }\delta \left(t-t^{\prime }\right)=\Delta t\delta_{kj}. \end{array}$$

The time-evolution operator is defined as:

(15) $$U\left(t,t_{0}\right)=\hat{T}\exp\left(-i\int_{t_{0}}^{t}H^{\prime }\left(t^{\prime }\right)dt^{\prime }\right).$$

We introduce the basis states [43]

(16) $$|i_{p}\rangle =\frac{{\left(\Delta B^{†}\left(t_{k}\right)\right)}^{i_{p}}}{\sqrt{i_{p}!\Delta t^{i_{p}}}}|\mathrm{vac}\rangle ,$$

where $i_{p}$, *p* integer, denotes the number of excitations present in the Fock state of the *k*th time interval $|i_{p}\rangle$. Writing Equation (10) in the basis of the noise operators enables us to define a discretized time-evolution operator U(Δt) where we may drop the time ordering $\hat{T}$ for equidistant time steps $\Delta t=t_{k+1}-t_{k}$, where $t_{k}$ denotes the *k*th time step:

(17) $$\begin{array}{c} U\left(t_{k+1},t_{k}\right)=\exp\left[i\sqrt{\Gamma }\Delta B\left(t_{k}\right)c^{†}-i\sqrt{\Gamma }\Delta B^{†}\left(t_{k}\right)c\right] \end{array}$$

for $k\in [0,N_{T}-1]$ as integer of the time steps. With this, we can use the QSSE operators defined in Equation (16) as the basis for the numerical non-Markovian time evolution.

Please note that the corresponding Hilbert space scales with the integration time and thus becomes very large. In the next section, we present an algorithm which preserves both the spatial and temporal entanglement of such a large system efficiently during time evolution. The algorithm adapts the structure of the construction of the MPS to enable a more efficient computation of the time-evolution. Here, we exploit the initially uncorrelated state of system and vacuum reservoir in such a way that the numerically costly swapping of MPS-bins is reduced significantly.

### 3.3. Algorithm

To compute the time evolution with tMPS, we expand the state vector into an MPS. The total wave vector ${|\psi \rangle }_{\mathrm{tot}}$ consists of the system wave vector ${|\psi \rangle }_{\mathrm{sys}}$ and the reservoir wave vector ${|\psi \rangle }_{\mathrm{res}}$:

(18) $$\begin{array}{c} {|\psi \rangle }_{\mathrm{tot}}={|\psi \rangle }_{\mathrm{sys}}⊗{|\psi \rangle }_{\mathrm{res}}. \end{array}$$

Written in the basis defined in Equation (16), it reads as:

(19) $$\begin{array}{cc} {|\psi \rangle }_{\mathrm{tot}}=\sum_{\overset{n_{1}\cdots n_{N}}{m_{1}\cdots m_{N_{T}}}} & c_{n_{1}\cdots n_{N},k_{1}\cdots k_{N_{T}}}\left(|n_{1}\cdots n_{N}\rangle ⊗|m_{1}\cdots m_{N_{T}}\rangle \right) \end{array}$$

with the complex coefficients $c_{n_{1}\cdots n_{N},m_{1}\cdots m_{N_{T}}}$ expanded into tensors *A*:

(20) $$\begin{array}{cc} c_{n_{1}\cdots n_{N},m_{1}\cdots m_{N_{T}}}=A_{n_{1}}^{\alpha_{n_{1}}}A_{n_{2}}^{\alpha_{n_{2}},\alpha_{n_{2}}^{\prime }} & \cdots A_{n_{N}}^{\alpha_{n_{N}},\alpha_{n_{N}}^{\prime }}\\ A_{m_{1}}^{\alpha_{m_{1}},\alpha_{m_{1}}^{\prime }}A_{m_{2}}^{\alpha_{m_{2}},\alpha_{m_{2}}^{\prime }} & \cdots A_{m_{N_{T}}}^{\alpha_{m_{N_{T}}}} \end{array}$$

where the index $n_{i}$ is the physical index of the *i*th site in the many-body system and $m_{k}$ the index of the state of the reservoir at the *k*th time step.

**Figure 2:**
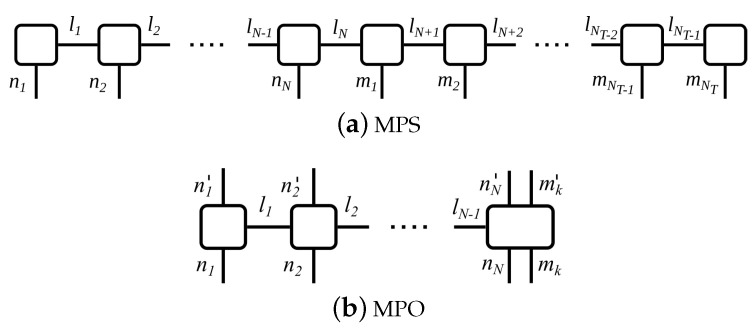
Block diagram for the time evolution of an open quantum system. Each tensor is depicted as a box, while the lines correspond to the indices of the respective tensor. Connecting lines between boxes indicate shared indices of the tensors. (**a**) MPS of the open quantum system: $n_{i}$ labels the site indices of the many-body system, while $m_{k}$ labels the time bins, and $l_{1}\cdots l_{N_{T-1}}$ labels the link indices. Initially, all system tensors are placed on the left in the MPS, followed by the time bins. During the time evolution, the system bins must be moved through the MPS to the right, a numerically very demanding procedure. (**b**) MPO for the time evolution of the dissipative many-body system where exemplary the last site is subject to dissipation. Please note that the MPO affects the entire many-body system, but only the reservoir time bin of the present time step.

Equation (20) contains the information about the system as well as of the state of the reservoir at every time step. They consist of *N* respectively $N_{T}$ connected tensors, where $N_{T}=\frac{T}{\Delta t}$ is the total number of time steps and *N* the number of sites in the many-body system. Thus, every matrix $A_{n_{i}}$ describes the state of the single sites as subsystems of the many-body system, while the matrices $A_{m_{k}}$ contain the information of the state of the reservoir at one time step. Thus, each tensor carries one physical index which is also called site index, and one or several link indices $\alpha_{n_{i}}$, $\alpha_{m_{k}}$. Figure 2a depicts this decomposition in the form of a block diagram.

Using the form in Equation (20) allows not only for the preservation of the state of the reservoir at every time step, but more importantly for the efficient truncation of the Hilbert space: The singular values of the decomposed wave vector matrices are related to the von Neumann entropy S(ρ) of the density operator ρ=|ψ〉〈ψ| which serves as a quantum mechanical measure of entanglement: if |ψ〉=|AB〉 is a composite quantum system consisting of the subsystems *A* and *B*, the entropy of entanglement measures the entanglement between the two subsystems and is expressed as [5,78]

(21) $$S_{A|B}\left(\rho_{A}\right)=S_{A|B}\left(\rho_{B}\right)=-\sum_{i}^{r}\lambda_{i}\log_{2}\lambda_{i},$$

where $\rho_{A}$,$\rho_{B}$ are the reduced density operators of the subsystems *A*, *B* respectively, *r* is the Schmidt rank and the $\lambda_{i}$ are the singular values of the decomposition between the two subsystems.

Thus, the singular values represent the entanglement between the many-body system, between reservoir and spin chain as well as between the state of the reservoir at different time steps. Truncating their entries during the decomposition process, thus setting them to zero below a given threshold, reduces the Hilbert space efficiently while losing only the paths with negligible probabilities. See [56,57,64,79,80,81,82,83,84] for detailed introductions to matrix-product states (See also the tensor network library website https://itensor.org/).

The time-evolution operator $U(t_{k+1},t_{k})$ in (17) is expanded accordingly into a matrix-product operator (MPO), cf. Figure 2b for the corresponding block diagram. Operator sums in the matrix exponential, which often occur in the computation of many-body systems, may be approximated using the Suzuki-Trotter decomposition, usually of second or third order [62,63]. Please note that the MPO affects all sites of the many-body system, but only the *k*th time step of the reservoir.

**Figure 3:**
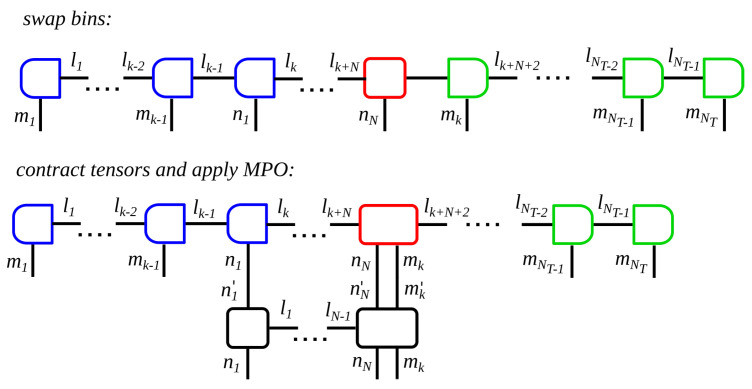
Block diagram of an algorithm with MPS for open quantum systems where one site of the many-body system is subject to dissipation. The diagram demonstrates the calculation of the *k*th time step. Blue boxes indicate left-orthogonality of the tensors, while green boxes indicate right-orthogonality and the red box marks the position of the orthogonality center of the MPS. The MPS contains the wave vector of the many-body system and of the reservoir. One time step is computed by applying the MPO on the many-body system, where the present state of the reservoir is contracted into the dissipative site. This step is illustrated in the lower figure and consists of contracting all tensor over their link indices while keeping the relevant site indices. Afterwards, the tensors are decomposed, and the present time bin must be swapped through the MPS to the left of the many-body system.

To explain the principle, we assume a many-body system which is dissipatively coupled to a reservoir at its last site. At the start of the time evolution, all system tensors are placed on the left end of the MPS, followed by all future time steps, cf. Figure 2a. To compute the *k*th time step, we contract the *N*th chain bin, the *k*th time bin initialized in a vacuum state and apply the MPO according to Equation (1), which means we multiply the MPO into the MPS. Afterwards, the *k*th time bin is moved to the left end of the MPS. With this, all following time steps may be computed accordingly. Figure 3 depicts the corresponding block diagram.

Please note that in order to swap the places of two tensors in one MPS, they need to exchange the link indices containing the entanglement information to the rest of the chain, which creates the need to contract and re-decompose them—otherwise the entanglement between the subspaces would not be preserved correctly [56]. This is no limitation in the case of few-level physics, as the system part of the MPS is small, as it usually holds that N=1. In case of many-body systems, however, this procedure becomes numerically very demanding due to the increasing size of the systems and also due to the strong entanglement growth within a many-body system during time evolution [67], and with this, the Schmidt values grow in number as well as in size—and may not be neglected during the truncation procedure. Subsequently, the number of non-zero entries on the system tensors grows strongly and so does computation effort. These are severe limitations which quickly make this numerical method less efficient than other standard methods for the time-evolving of open many-body systems and limit the complexity of the structured bath.

We present here an algorithm which overcomes this limitation as it operates without swapping the position of the bins. It makes use of two physical properties of the dynamics: First, the fact that future time bins are not yet entangled with the system dynamics, and secondly, that for Markovian interaction, only the present state of the bath matters. Thus, we only need to include the present state of the reservoir in the chain and may lose the information of the state of the reservoir in previous time steps.

**Figure 4:**
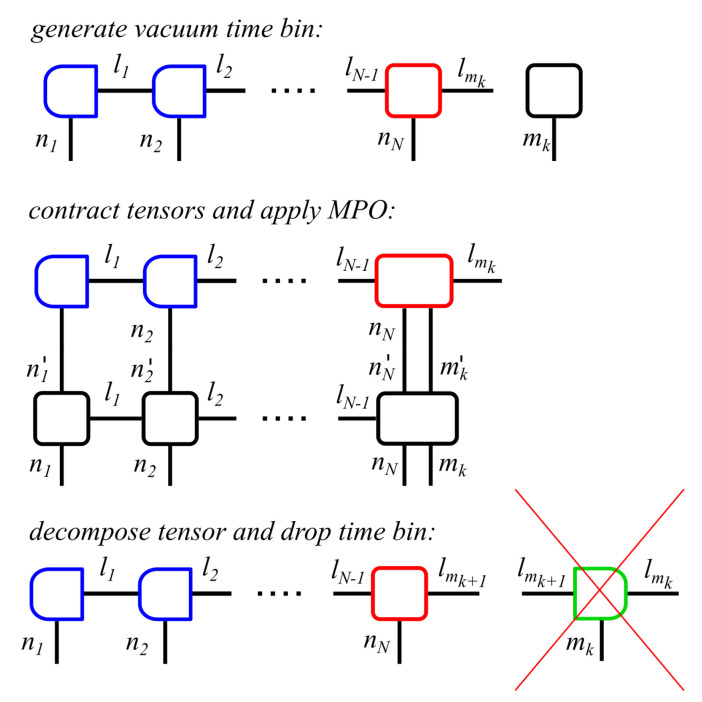
Block diagram of an efficient algorithm for open quantum systems where one site of the system is subject to dissipation. The diagram demonstrates the calculation of the *k*th time step. Blue boxes indicate left-orthogonality of the tensors, while green boxes indicate right-orthogonality and the red box marks the position of the orthogonality center of the MPS. One time step is computed as follows: the present state of the reservoir is modeled with a single time bin initialized in the vacuum state, while the MPS only contains the system bins. The time bin is multiplied into the dissipative site of the many-body system and the MPO is applied according to Equation (1). Afterwards, the tensors are being decomposed and the time bin is dropped, including its link to the past.

We start with an MPS which contains the systems bins, one for each site of the many-body system, followed by one time bin in vacuum state representing the present state of the reservoir. To compute one time step, we contract the present time bin with the last site and apply the MPO. We decompose the two tensors and only keep the tensor describing the state of the last site of the many-body system, thus we lose the information of the state of the bath as well as the entanglement of the state of the bath with the its own past. We generate a new vacuum bin—as it represents the future state of the bath, it is not yet correlated with the system, thus it may be initialized apart from the many-body system - multiply it into the last site, and continue with the application of the MPO, cf. Figure 4 for the block diagram.

With this, the swapping of the reservoir bins through the MPS is avoided, which results in a significant reduction of the computational effort and enables the calculation of the dynamics of larger many-body systems possibly up to the thermodynamical limit—cf. Section 5 for application examples.

## 4. Modeling Non-Markovian System—Reservoir Interaction

### 4.1. Model

**Figure 5:**
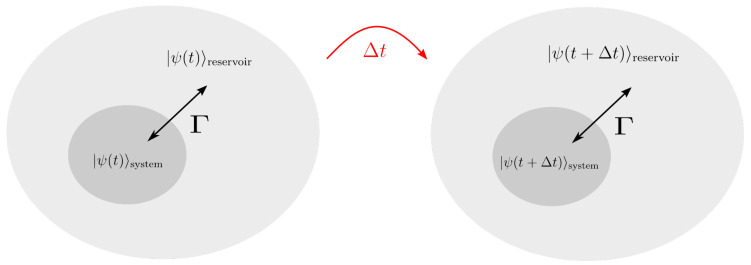
Sketch of an open quantum system with non-Markovian type of interaction. The total system consists of a microscopic region ${|\psi (t)\rangle }_{\mathrm{sys}}$ which couples to its surrounding environment or reservoir ${|\psi (t)\rangle }_{\mathrm{res}}$ with a coupling strength Γ. Contrary to the Markovian case, the state of the reservoir at the time t+Δt remains influenced by the interaction with the system which has occurred during the time step Δt, thus ${|\psi (t+\Delta t)\rangle }_{\mathrm{res}}\neq {|\psi (t)\rangle }_{\mathrm{res}}$.

In many cases, the large separation of time scales between system and reservoir, which results in the assumption of an instant recovery of the reservoir from the interaction with the system, does not hold, cf. Figure 5. This results in non-Markovian effects which occur in a broad range of contexts for instance in quantum optics, solid state physics, quantum chemistry and many more [6,7,11,12,13]—see [15] for an excellent review of the topic.

Again, the total Hamiltonian reads as defined in Equation (2), where the properties of the free evolution of the system $H_{\mathrm{sys}}$ and of the reservoir $H_{\mathrm{res}}$ are left unchanged. However, the information backflow from the reservoir introduces non-timelocal contributions into the interaction part $H_{\mathrm{int}}$

(22) $$H_{\mathrm{int}}=\sum_{i=1}^{N}\int d\omega \left(G\left(\omega \right)b^{†}\left(\omega \right)c^{-}+h.c.\right)$$

which are included in a frequency dependence of the coupling element G(ω). Translating into the tMPS formalism the fact that the present state of the reservoir is influenced by interactions with the system which have occurred in the past, this implies that for calculating the dynamics of the total system, the information backflow from the reservoir of previous time steps needs to be taken into account.

In the following, we demonstrate an efficient algorithm for a non-Markovian time evolution. Analogous to the Markovian case, we use the uncorrelated initial state between system and reservoir for constructing an MPS-form which enables efficient movement of bins during time-evolution. For simplicity, we demonstrate this using a model where one previous time step needs to be taken into account - generalizations to more previous time steps may be made without conceptional difficulty. This algorithm not only demonstrates the principle ideas very well, it also describes the dynamics of some open systems, as demonstrated in Section 5 further below.

### 4.2. Algorithm

**Figure 6:**
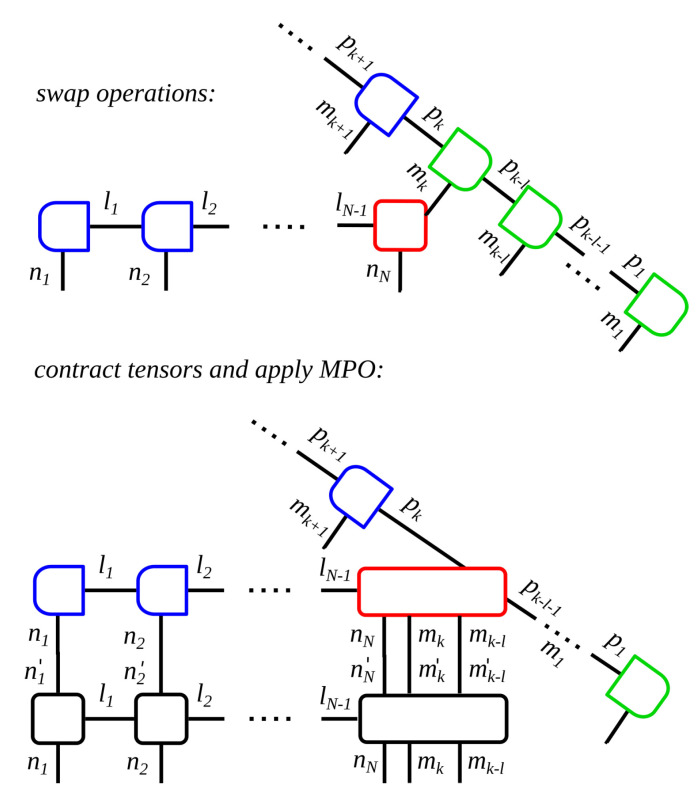
Block diagram for the computation of one time step. Blue boxes indicate left-orthogonality of the tensors, while green boxes indicate right-orthogonality and the red box marks the position of the orthogonality center of the MPS. The many-body system MPS and the reservoir MPS are connected at the *k*th time bin $m_{k}$. Also, the past time bin $m_{k-l}$ has been brought next to them with swapping operations. For the application of the MPO, the *N*th chain bin, the present and feedback time bin $m_{k}$ and $m_{k-l}$ are contracted and the chain MPO is multiplied into the MPS. Afterwards, the tensors are decomposed and moved back to their original position in the chain.

Figure 6 depicts the block diagram algorithm for our system with non-Markovian dynamics. We assume that the last site of a many-body system is in interaction with the reservoir. To compute the *k*th time step, we contract the *N*th chain bin, the *k*th time bin initialized in a vacuum state and the $t_{k-l}$th bin containing the feedback signal, and apply the time-evolution operator $U(t_{k+1},t_{k})$ as described in Section 3.

In case of non-Markovian interaction, time evolving the total state vector as a single MPS becomes numerically very demanding, as time bins from the past have to be moved back and forth through the chain bins additionally to moving the chain through the reservoir during every time step. In case of an open many-body system, the growing spatial entanglement within the system significantly additionally contributes to this, making larger *N* difficult to access.

To overcome this limitation, we construct a two-dimensional MPS. As the reservoir is in a vacuum state initially and not yet entangled with the spin chain, the wave vector both of system and bath may be expanded separately at the start of the time evolution. The wave vector reads as:

(23) $$\begin{array}{cc} |\psi (t_{k})\rangle = & \sum_{\overset{n_{1}\cdots n_{N}}{=0,1}}c_{n_{1}\cdots n_{N}}|n_{1}\cdots n_{N}\rangle \\ \; & ⊗\sum_{m_{1}\cdots m_{N_{T}}}c_{m_{1}\cdots m_{N_{T}}}|k_{1}\cdots k_{N_{T}}\rangle \end{array}$$

where both complex coefficients are expanded into products of tensors *A*:

(24) $$\begin{array}{cc} c_{n_{1}\cdots n_{N}} & =A_{n_{1}}A_{n_{2}}\cdots A_{n_{N}} \end{array}$$

(25) $$\begin{array}{cc} c_{m_{1}\cdots k_{N_{T}}} & =A_{m_{1}}A_{m_{2}}\cdots A_{m_{N_{T}}} \end{array}$$

**Figure 7:**
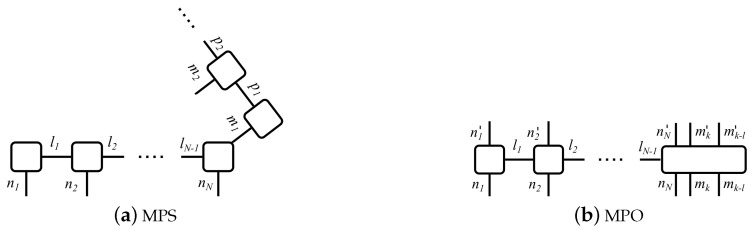
Block diagram of MPS and MPO of a many-body system in non-Markovian interaction with a reservoir. (**a**) the MPS is 2-dimensional and consists of one MPS with the system bins labeled with the site indices $n_{i}$ and the link indices $l_{i}$, and one MPS containing the reservoir bins labeled with the site indices $m_{k}$ and the link indices $p_{k}$. The two MPS are stuck together at the *N*th system bin where the interaction occurs. (**b**) MPO for the computation of the time evolution according to Equation (1). Please note that it affects all system bins, yet only the present time bin $m_{k}$ and relevant time bin describing the past state of the reservoir $m_{k-l}$, where l∈N denotes the number of time steps between present and relevant past state of the reservoir.

Figure 7 depicts the block diagram of the MPS and the MPO. To preserve the entanglement between the two subspaces during time evolution, we stick them together at the *N*th chain bin and the *k*th time bin, where the interaction between the many-body system and the bath occurs. We keep this connection throughout the entire time evolution and thus preserve the entanglement between the two subspaces.

With this, the number of swapping operations reduces significantly. The time bin of the past time step only must be moved through the reservoir MPS, where the entanglement is usually well below the one in the chain. In addition, only the *N*th chain bin must be moved through the reservoir MPS during time evolution, not the entire chain. After applying the MPO, we decompose the tensor again, shift the bins back to their original position in the chain, move and contract the bins of the (k+1)th time step and so forth. Care must be taken to keep the orthogonality center at the right position to preserve the entanglement information correctly. Its position is indicated by the red box in Figure 6.

This construction enables us to avoid the swapping of the time bins through the many-body part of the MPS, which would be even more costly in case of one (or more) time bins of the past are needed to compute the present time step. Please note that the two MPS may be initialized separately because of the initial uncorrelated state of system and reservoir. Also note that due to the transformation into the QSSE-picture, we assume the reservoir of the present time step to be in the vacuum state. The algorithm also deals efficiently with the extension of the structure to more individual reservoirs on other sites. We remark that however, the number of sites coupling to the same reservoir is limited due to the necessary contraction operations.

## 5. Application Examples

### 5.1. A Dissipative Spin Chain with Markovian Interaction

As a paradigmatic many-body system, we model an open, isotropic Heisenberg spin-1/2 chain with nearest-neighbor interaction. Thus, in Equation (2), $H_{\mathrm{sys}}$ reads as:

(26) $$H_{\mathrm{sys}}=\sum_{i=1}^{N}\omega_{0}\sigma_{i}^{+}\sigma_{i}^{-}+\sum_{i=1}^{N-1}J\left(\sigma_{i}^{x}\sigma_{i+1}^{x}+\sigma_{i}^{y}\sigma_{i+1}^{y}+\sigma_{i}^{z}\sigma_{i+1}^{z}\right)$$

The first term models the free evolution of *N* single spin systems, where $\omega_{0}$ governs the free evolution of each single site and $\sigma_{i}^{\pm }=\sigma_{i}^{x}\pm i\sigma_{i}^{y}$ creates/annihilates a fermionic excitation in the *i*th two-level system which is equivalent to a flip of the spin on site *i* [76,85,86,87,88,89]. The second term describes the isotropic Heisenberg spin chain, a chain with *N* single sites and a three-dimensional nearest-neighbor interaction in *x*, *y* and *z* direction, where $\sigma^{k}$, k∈x,y,z represent the Pauli matrices interacting with strength *J*. We couple the last site to a vacuum reservoir which may for instance be created by the interaction with an infinite waveguide. On the first site of the chain, we drive it coherently with a laser with the continuous wave pump field Ω which we apply on the first site of the chain, thus we add the following term to Equation (26):

(27) $$H_{\mathrm{pump}}=\Omega \left(\sigma_{1}^{+}e^{-i\omega_{L}t}+\sigma_{1}^{-}e^{i\omega_{L}t}\right).$$

We transform the full Hamiltonian $H_{\mathrm{tot}}$ in Equation (2) into the QSSE-picture as described in Section 3.2 and solve the dynamics for |ψ(t)〉 as described in Section 3.3. The resulting time-evolution operator reads:

(28) $$\begin{array}{cc} U\left(t_{k+1},t_{k}\right)=\exp[i\Omega \left(\sigma_{1}^{+}+\sigma_{1}^{-}\right)\Delta t+i\sum_{i=1}^{N-1}J(\sigma_{i}^{x}\sigma_{i+1}^{x}+ & \sigma_{i}^{y}\sigma_{i+1}^{y}+\sigma_{i}^{z}\sigma_{i+1}^{z})\Delta t\\ \; & +i\sqrt{\Gamma \Delta t}\Delta B\left(t_{k}\right)\sigma_{N}^{+}-i\sqrt{\Gamma \Delta t}\Delta B^{†}\left(t_{k}\right)\sigma_{N}^{-}] \end{array}$$

As the chain is driven by the pump field on its left end and displays a Lindblad type of decay on its right end, the figure of merit is the spin current through the chain. It is defined as the average current per site, thus as:

(29) $${\langle j\left(t\right)\rangle }_{\mathrm{rel}}=\frac{1}{N-1}\sum_{i=1}^{N-1}\langle j_{i}\left(t\right)\rangle ,$$

where $\langle j_{i}\left(t\right)\rangle$ denotes the spin current between the *i*th and i+1th site in the chain.

**Figure 8:**
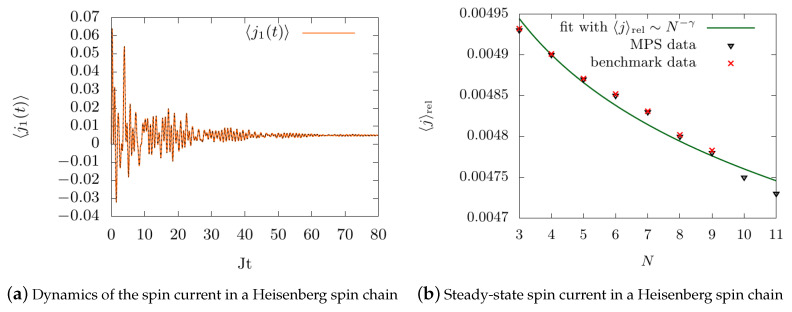
Algorithm benchmark with the full master equation: (**a**) Time dynamics of the spin current between the first and second site in a chain with N=4 sites in a chain initialized in the Neel state, thus |↑↓↑↓〉. Clearly, initially the current oscillates irregularly and finally equilibrates out to a non-equilibrium steady state (NESS). Please note that the curve is plotted twice, as this figure furthermore serves as a benchmark using the full solution for |ψ(t)〉 with the Lindblad master equation (black dotted line). (**b**) Relative current ${\langle j\rangle }_{\mathrm{rel}}$ through the chain as a function of the number of sites *N* of the many-body system (black triangles). The data is fitted with a power law function, where we obtain the parameter γ=0.01±0.0006, indicating a superdiffusive behavior corresponding to [30]. We demonstrate the benchmark for small system sizes using the full solution of the master equation (red crosses).

Figure 8 demonstrates the benchmark of the algorithm with the full solution of the Lindblad master equation. In Figure 8a, the time dynamics of the spin current between the first and second site in a chain with N=4 sites initialized in the Neel state |↑↓↑↓〉 is depicted exemplary. Clearly, initially the current oscillates irregularly and finally equilibrates out to a non-equilibrium steady state (NESS), where the current between all sites takes on the same value. Please note that the curve is plotted twice, as Figure 8 furthermore serves as a benchmark using the full solution for |ψ(t)〉 with the Lindblad master equation (black dotted line).

In recent years, there has been a growing interest in transport properties of the spin chains, especially of the paradigmatic model of the Heisenberg chain. In this work, the chain is coupled to reservoirs at both ends and, using a full Markovian interaction, it is incoherently driven into a NESS [18,19,32,65,66,67,68,69,70,71,72,90,91,92]. For the weak driving regime, anomalous transport properties have been demonstrated [30,65,93]. Here, it has been shown that for the isotropic case, the current displays superdiffusive behavior, thus it holds that ${\langle j\rangle }_{\mathrm{rel}}∼N^{-\gamma }$ with 0<γ<1. This is in contrast to the diffusive case, where it holds that γ=1 - thus in the superdiffusive case, the current decreases slower with increasing system than in the diffusive case.

In Figure 8b, we measure the relative current ${\langle j\rangle }_{\mathrm{rel}}$ through the chain as a function of the number of sites *N* of the many-body system (black triangles). The data is fitted with a power law function (green line), where we obtain the parameter γ=0.01±0.0006, indicating a superdiffusive behavior. We demonstrate the benchmark for small system sizes using the full solution of the master equation (red crosses).

Interestingly, despite the coherent driving we apply in this model, this corresponds to the result for an incoherently driven chain modeled with a Lindblad master equation [30], where the current also displays superdiffusive behavior in the weak driving regime. After providing this benchmark, we will in the following section demonstrate the full power of the algorithm for the more general, yet numerically more complex case of a non-Markovian system–reservoir type of interaction.

### 5.2. An Open Spin Chain in a Semi-Infinite Waveguide

In the second scenario, we consider the isotropic Heisenberg spin chain described by Equation (26) to be embedded in a non-Markovian, structured reservoir. This consists of a semi-infinite waveguide [88,89,94,95,96,97,98,99,100,101] where the closed end is modeled by a mirror in distance *L* to the spin chain. Part of the excitation emitted from the chain will then be reflected by the mirror and interacts with the system for a second time after the delay time τ. This means that the coupling term in Equation (22) is sinusoidal frequency dependent and reads as $G_{fb}\left(\omega \right)=g_{0}\sin\left(\frac{\omega L}{c_{0}}\right)$, where $c_{0}$ is the phase velocity in the waveguide. This G(ω) introduces non-timelocal coupling into the system–reservoir dynamics. This coherent, feedback-based non-Markovian system–reservoir coupling is known from and predominantly studied in atom-molecular-optics and cavity-QED [43,97,99,100,101,102,103,104,105]. It introduces quantum interferences into the system dynamics and has been well investigated for the model of a single few-level emitter [43,75,105,106,107,108], where a stabilization of quantum coherence due to interference effects between incoming and outgoing probability waves [109,110] is observed. In particular, Rabi oscillations in the single-excitation regime have been predicted [111], which emerge if the roundtrip-time τ is a multiple of the inverse of the cavity-emitter coupling g/(2π). They are up-to-now limited to the single-excitation and single-emitter regime.

Using the proposed algorithm, we extend the investigation to a many-body system. By doing so, we demonstrate that these limitations can be lifted and that excitation trapping as well as feedback-induced stabilization of coherent oscillations are of general character and apply also to strongly correlated many-body systems such as the Heisenberg chain. Using our proposed algorithm, we are additionally able to demonstrate their existence for large system sizes.

We transform the full Hamiltonian $H_{\mathrm{tot}}$ described with Equation (2) where $H_{\mathrm{sys}}$ is given by Equation (26) and $H_{\mathrm{int}}$ by Equation (22) into the picture of the quantum stochastic Schrödinger equation. The resulting time-evolution operator reads:

(30) $$\begin{array}{cc} U(t_{k+1},t_{k})=\exp[\; & i\sum_{i=1}^{N-1}J\left(\sigma_{i}^{x}\sigma_{i+1}^{x}+\sigma_{i}^{y}\sigma_{i+1}^{y}+\sigma_{i}^{z}\sigma_{i+1}^{z}\right)\Delta t\\ \; & +\sqrt{\Gamma \Delta t}\left(\Delta B_{N}\left(t_{k}\right)-\Delta B_{N}\left(t_{k-l}\right)e^{i\phi }\right)\sigma_{N}^{+}-\sqrt{\Gamma \Delta t}\left(\Delta B_{N}^{†}\left(t_{k}\right)-\Delta B_{N}^{†}\left(t_{k-l}\right)e^{-i\phi }\right)\sigma_{N}^{-}] \end{array}$$

Here, $t_{k}$ denotes the *k*th time step, while $t_{k-l}=\left(k-l\right)\Delta t$ denotes the time delayed by $\tau =2L/c_{0}$ and $\phi =\omega_{0}\tau$ denotes the feedback phase. As initial state, the last spin is assumed to be in excited state while all remaining sites are initialized in ground state.

As has been demonstrated for single few-level emitters, we observe population trapping when subjecting the chain to coherent self-feedback. This means that the initial excitation within the chain dissipates partially into the reservoir until this process is stopped by the interaction with the feedback signal and modifies the dissipative coupling due to quantum interferences. In consequence, after a parameter-dependent time $T_{c}$, the system–reservoir interaction reaches a steady-state and traps dynamically the remaining excitation within the chain.

The conditions for population trapping depend on two parameters: The delay time τ and the feedback phase ϕ. Importantly, the two parameters are not independent in this setup, as it holds that $\phi =\omega_{0}\tau$. For the case of the many-body system under feedback, the conditions for the trapping to take place differ significantly from the case of a single two-level system. In case of a single two-level system, population trapping only occurs at $\phi =\omega_{0}\tau =2\pi n$ with *n* integer, i.e., in the interval [0,2π) only one phase allows population trapping. This is significantly different in our system. We find in the interval [0,2π) several conditions for $\phi_{c}$ which leads to population trapping, and the number of possible $\phi_{c}$ depends in strong contrast to the single two-level emitter case on τ. The reason for this is the interaction dynamics within the chain which imposes new conditions for the critical feedback phase $\phi_{c}$. Strikingly, we find that despite the complex many-body dynamics within a Heisenberg chain with N>2, the number of $\phi_{c}$ within one 2π-interval $N_{\phi_{c}}$ is equal to the number of interacting sites within the chain, $N_{\phi_{c}}=N$, which allows for a partial, non-invasive characterization of the spin chain, see [112].

**Figure 9:**
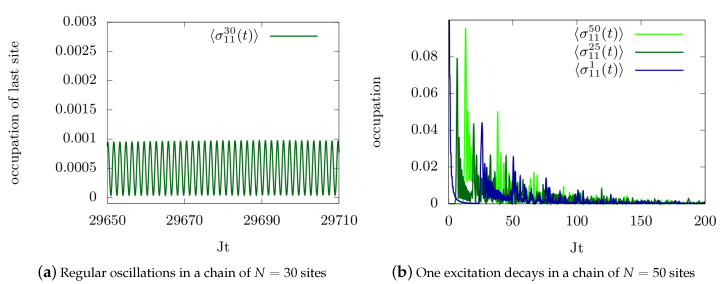
Dynamics of the time-dependent occupation densities ${\langle \sigma_{11}\left(t\right)\rangle }^{i}$ in a Heisenberg chain of different lengths *N* and for different trapping conditions $\phi_{c}$, $\tau_{c}$. (**a**) Dynamics for population trapping: Regular and periodic oscillations in a chain of N=30 sites. Parameters for this plot are Γ=1.5, J=0.1. (**b**) Dynamics without population trapping: Clearly, the initial state quickly dissipates into the environment and no excitation remains within the chain. Please note that we only plot a few selected sites in the chain. Parameters for this plot are Γ=0.24 and J=0.1.

Investigating the steady-state behavior for different feedback phases and time delays, we observe three possibilities: First, in the long-time limit all excitation of the chain is lost, second, all single site occupation densities in the chain are finite and constant, and third, the total excitation in the chain remains constant and finite but the densities $\langle \sigma_{11}^{i}\rangle$ oscillate. The first case is the rule, as most delay times and phases do not allow a non-trivial steady-state in combination with the quantum spin chain dynamics but will lead to a complete loss of excitation to the environment. The second case relates to almost all trapped states. Here, we find that each set of critical parameters $\phi_{c}$, $\tau_{c}$ may be characterized with one specific trapped state. The third case appears at degeneracy points, where two or more population trapping conditions $\phi_{c}$, $\tau_{c}$ hold. Here, highly non-trivial steady states are the result: Stabilized oscillations within the chain occur and a periodic, time-dependent steady-state is created. These steady states differ however in coherence and relative phase shifts between the trapped occupation densities ${\langle \sigma_{11}^{i}\rangle }_{tr}$ at different intersection points. This induced, synchronized and constant excitation within the chain although the system is open is an important result which also holds for more excitations in the chain and different initial states [112]. This holds for different decay strengths Γ and feedback delay times τ, as well as feedback phases ϕ, and is a generic feature of such a system. Importantly, our numerical method enables us to demonstrate that these oscillations do not only occur for small system sizes but also approaching the thermodynamic limit, as demonstrated in Figure 9a, which displays an example of a regular, time-reversible oscillation pattern for a chain length of N=30. This system behavior contrasts strongly with the case where no trapping conditions hold, as depicted in Figure 9b. It depicts the time-dependent occupation densities (blue and green) of a Heisenberg spin chain with N=50 sites where the occupation of the last site dissipates into vacuum, thus for an Markovian type of interaction. Clearly, the initial state quickly dissipates into the environment and no excitation remains within the chain. This figure also demonstrates the power of the algorithm for larger system sizes in the Markovian regime.

## 6. Conclusions

In this paper, we presented an efficient algorithm for the time evolution of open quantum many-body systems using matrix-product states (MPS) in the basis of the quantum stochastic Schrödinger picture which exploits the initially with the system uncorrelated vacuum reservoir for re-structuring the architecture of the MPS. We explained its applicability to systems with Markovian type of interaction, where only the present state of the reservoir needs to be taken into account. Furthermore, we demonstrated its extension to a non-Markovian type of interaction, where the information backflow from the reservoir needs to be taken into account. Afterwards, we presented the application of the algorithm using the Heisenberg spin chain as a paradigmatic example of a many-body system, and investigating two settings. As a benchmark example, we drove the chain with a constant pump field and measured the spin current through the chain. Secondly, we demonstrated the accessibility of larger systems with our algorithm by using it in order to extend the application of coherent self-feedback on a quantum many-body system and thus demonstrate the applicability for non-Markovian type of interaction with the reservoir.
